# Warming increases the sensitivity of seedling growth capacity to rainfall in six temperate deciduous tree species

**DOI:** 10.1093/aobpla/ply003

**Published:** 2018-01-17

**Authors:** Vikki L Rodgers, Nicholas G Smith, Susanne S Hoeppner, Jeffrey S Dukes

**Affiliations:** 1 Math and Science Division, Babson College, Wellesley, MA, USA; 2 Department of Biological Sciences, Texas Tech University, Lubbock, TX, USA; 3 Department of Forestry and Natural Resources, Purdue University, West Lafayette, IN, USA; 4 Purdue Climate Change Research Center, Purdue University, West Lafayette, IN, USA; 5 Department of Psychiatry, Harvard Medical School, Boston, MA, USA; 6 Department of Psychiatry, Massachusetts General Hospital, Suite, Boston, MA, USA; 7 Department of Biological Sciences, Purdue University, West Lafayette, IN, USA; 8 Department of Biology, University of Massachusetts Boston, Boston, MA, USA

**Keywords:** Boston-Area Climate Experiment (BACE), carbon, climate change, drought, leaf area, nitrogen, tree seedlings, warming

## Abstract

Predicting the effects of climate change on tree species and communities is critical for understanding the future state of our forested ecosystems. We used a fully factorial precipitation (three levels; ambient, −50 % ambient, +50 % ambient) by warming (four levels; up to +4 °C) experiment in an old-field ecosystem in the northeastern USA to study the climatic sensitivity of seedlings of six native tree species. We measured whole plant-level responses: survival, total leaf area (TLA), seedling insect herbivory damage, as well as leaf-level responses: specific leaf area (SLA), leaf-level water content (LWC), foliar nitrogen (N) concentration, foliar carbon (C) concentration and C:N ratio of each of these deciduous species in each treatment across a single growing season. We found that canopy warming dramatically increased the sensitivity of plant growth (measured as TLA) to rainfall across all species. Warm, dry conditions consistently reduced TLA and also reduced leaf C:N in four species (*Acer rubrum*, *Betula lenta*, *Prunus serotina*, *Ulmus americana*), primarily as a result of reduced foliar C, not increased foliar N. Interestingly, these conditions also harmed the other two species in different ways, increasing either mortality (*Populus grandidentata*) or herbivory (*Quercus rubra*). Specific leaf area and LWC varied across species, but did not show strong treatment responses. Our results indicate that, in the northeastern USA, dry years in a future warmer environment could have damaging effects on the growth capacity of these early secondary successional forests, through species-specific effects on leaf production (total leaves and leaf C), herbivory and mortality.

## Teaser

Using a fully factorial precipitation by warming experiment in an old-field ecosystem in the northeastern USA we studied the climatic sensitivity of seedlings of six native tree species. Warm and dry conditions suppressed seedling growth, but affected species differently by increasing mortality, enhancing rates of herbivory or decreasing foliar carbon uptake. Our results indicate that, in the northeastern USA, dry years in a future warmer environment could have damaging effects on the growth capacity of early secondary successional forests, through species-specific effects on leaf production, herbivory and mortality.

## Introduction

Climate change is warming air temperatures and shifting precipitation patterns (IPCC 2013; Dai 2013), which will increasingly affect the growth of tree seedlings, leading to long-term changes in the composition and productivity of forests. Many of these changes will be consequences of species- and age-specific sensitivities (Martínez-Vilalta and Lloret 2016). Already, climatic shifts are modifying forest structure and function and have resulted in widespread forest die-back (van Mantgem *et al.* 2009; Allen *et al.* 2010; Anderegg *et al.* 2016). Predicting the effects of climate change on species composition and quantifying how individual tree species respond to climate variability is critical for understanding the future state of our forested ecosystems (Walther *et al.* 2002; Parmesan and Yohe 2003; Chen *et al.* 2011; Sittaro *et al.* 2017).

Studies have shown that plant tolerances and responses to climatic conditions change over the course of development (Cavender-Bares and Bazzaz 2000; Fisichelli *et al.* 2014). During early development, tree growth is particularly sensitive to external perturbations because the still-shallow root systems have limited access to deep reserves of soil water (Niinemets 2010; Wright *et al.* 2013). Simple scaling frameworks that fail to account for developmental stages often miss the fact that different age classes use different mechanisms to tolerate drought or warming conditions (Cavender-Bares and Bazzaz 2000). Although some adult trees may be predicted to persist through dramatic shifts in the climate, tree species ranges will eventually contract where tree regeneration fails (Bell *et al.* 2014).

Field data from multilevel temperature and precipitation experiments can be used to refine or benchmark the processes within current-generation climate models (Smith *et al.* 2014). The use of plant functional traits within climate models can improve predictions of species distributions (Kearney and Porter 2009; Webb *et al.* 2010; Van Bodegom *et al.* 2012; Scheiter *et al.* 2013). Flexible trait-based models that characterize organisms in terms of their multiple biological attributes allow for changes in the trait distribution (usually measured at the individual level and used comparatively across species) and associated modifications to community composition or ecosystem function to be predicted across time and space (Webb *et al.* 2010). Whereas the majority of field studies infer changes in trait values by looking across natural environmental gradients that confound differences in populations and species with the environmental variable of interest, in this study we measured functional plant traits under the factorial manipulation of different levels of temperature and precipitation together within a single community.

Total leaf (surface) area (TLA) and specific leaf area (SLA: leaf area per unit leaf mass) are key indicators of a plant’s carbon and water balance (Parkhurst and Loucks 1972), serve as important plant functional traits and are well known to vary with climate (Whittaker and Niering 1975). To survive changes in temperature and precipitation regimes, long-lived plants must either be capable of tolerating the new conditions or change physiologically and/or morphologically to accommodate them. Warmer conditions often promote photosynthesis and net primary productivity (Rustad *et al.* 2001; Wu *et al.* 2011), unless the warming exceeds metabolic optima or causes soil moisture stress (De Valpine and Harte 2001; Niu *et al.* 2006; Sage *et al.* 2008; Zhao *et al.* 2013).

In general, drier conditions typically suppress TLA, which reduces water loss, and increase biomass allocation to roots, which increases water uptake capacity (Smith and Huston 1989; Aspelmeier and Leuschner 2006; Erice *et al.* 2010). A number of studies have found reductions in SLA under drought conditions (Ibrahim *et al.* 1997; Thomas 2000; Valladares and Sánchez-Gómez 2006; Poorter *et al.* 2009), which is suggestive of an enhanced ability to avoid wilting (Maximov 1929), increasing drought resistance (Valladares and Sánchez-Gómez 2006; Greenwood *et al.* 2017). Warmer temperatures are likely to enhance drought responses by reducing soil moisture (supply) and increasing atmospheric water demand.

Changes in temperature and precipitation regimes are also known to directly influence rates of plant nutrient uptake and allocation of nutrients to leaf tissue (BassiriRad 2000; Reich and Oleksyn 2004). If photosynthetic carbon gain responds differently to these environmental changes than nitrogen uptake or allocation, leaf tissue chemistry could change. Interestingly, previous studies have found that warming can either increase (Nijs *et al.* 1996; Oleksyn *et al.* 2003; Rodgers *et al.* 2012) or decrease (Yin 1993; Dormann and Woodin 2002; Reich and Oleksyn 2004; Suseela *et al.* 2015) a plant species’ foliar nitrogen concentration. Altered foliar carbon and nitrogen concentrations can affect ecosystem-scale processes by influencing rates of photosynthesis, herbivore forage quality, plant litter chemistry and ultimately carbon and nutrient pathways (Shaver *et al.* 2001; Aerts *et al.* 2009).

In this study, we measured peak season whole plant- and leaf-level responses to warming and altered precipitation across a single growing season in the northeastern USA. Specifically, we measured changes in survival, TLA, leaf-level insect herbivory damage, SLA, leaf-level water content (LWC), and foliar carbon and nitrogen concentration in seedlings of six tree species: *Acer rubrum* (red maple), *Betula lenta* (sweet birch), *Populus grandidentata* (big-toothed aspen), *Prunus serotina* (black cherry), *Quercus rubra* (red oak) and *Ulmus americana* (American elm). Most of these species have large current and projected ranges that span much of the eastern and Midwestern USA (Prasad *et al.* 2007-ongoing; Iverson *et al.* 2008). However, *B. lenta* is restricted longitudinally, suggesting greater precipitation sensitivity, and *P. grandidentata* is restricted latitudinally, suggesting greater temperature sensitivity (Prasad *et al.* 2007-ongoing; Iverson *et al.* 2008). The tree seedlings in this study were grown within a high-light, old-field ecosystem, thereby simulating early secondary successional dynamics. Previous studies at this same experimental field site have found that the precipitation treatments altered heterotrophic soil respiration (Suseela *et al.* 2012), herbaceous plant water use efficiency (Rodgers *et al.* 2012) and tree seedling photosynthesis rates (Smith *et al.* 2016). In contrast, warming has had more limited direct effects, in part because of process acclimation. Warming suppressed the temperature sensitivity of heterotrophic (Suseela *et al.* 2012) and total soil respiration (Suseela and Dukes 2013), as well as soil nitrogen transformations (Auyeung *et al*. 2013), but also increased leaf nitrogen concentration in herbaceous plant tissue (Rodgers *et al.* 2012) and increased the intrinsic water use efficiency of tree seedlings (Smith *et al.* 2016). The combination of warming and dry conditions suppressed total herbaceous plant production, shoot production and plant species richness (Hoeppner and Dukes 2012), doubled the concentration of total tannins in *A. rubrum* leaf litter (Tharayil *et al.* 2011) and increased concentrations of other water-stress-related compounds in *Q. rubra* leaves (Suseela *et al.* 2015).

For this study we formulated hypotheses at the species, whole plant and leaf levels. We expected that responses of individual tree species could be inferred from their climatic niches (Prasad *et al.* 2007-ongoing; Iverson *et al.* 2008). We hypothesized that warmer temperatures combined with high levels of precipitation would favour growth of most species. We expected that treatments with drier soil conditions, either from reduced precipitation, warming or the combination, would reduce growth of most species, but added precipitation would counteract the negative effects of soil drying under warmed conditions. The species with the most limited climatic niches were *B. lenta* and *P. grandidentata*; their native ranges are more restricted to cooler and wetter regions (Prasad *et al.* 2007-ongoing; Iverson *et al.* 2008). We therefore hypothesized that these two species would be most negatively affected by (1) increased levels of warming, (2) reduced precipitation and (3) the combination of warming and reduced precipitation.

Our whole plant-level hypotheses were related to (i) tree seedling survival, (ii) estimated TLA and (iii) leaf herbivory damage. We expected warming to increase these variables under wet and ambient conditions, but decrease them under drier conditions.

We also tested the following leaf-level hypotheses: (i) LWC will decline across the gradient of increased warming and with reduced precipitation, and will be lowest with the combination of increased warming and drier conditions. (ii) SLA and (iii) foliar N will increase (lowering C:N ratio) with warming and with added precipitation, but will decline with the combination of increased warming and drier conditions.

We expected whole plant- and leaf-level responses to scale linearly with the magnitude of warming.

## Methods

### Study site and experimental design

This research was conducted at the Boston-Area Climate Experiment (BACE), a multifactorial climate manipulation experiment in an old-field ecosystem at the University of Massachusetts’ Suburban Experiment Station in Waltham, MA, USA (42°23′N, 71°13′W). The site experiences a mean annual temperature of 9.3 °C, a mean annual precipitation of 1194 mm year^−1^ (Auyeung *et al*. 2013), and features a loam topsoil over a gravelly sandy loam subsoil.

The BACE utilizes a full-factorial, split-plot, randomized block design with the precipitation treatment as the whole-plot factor and the temperature treatments as subplot factors nested within the precipitation treatment. Four levels of warming crossed with three levels of precipitation produced 12 different climate regimes, each of which was replicated three times for a total of 36 plots (2 m × 2 m in size). The four levels of warming were unwarmed (ambient temperature), low warming (+~1.3 °C multi-year average), medium warming (+~2.7 °C multi-year average) and high warming (target of +4.0 °C), and the three levels of precipitation were ambient rainfall, 50 % reduced rainfall (dry) and 50 % increased rainfall during the growing season (wet). Warming values achieved varied by season, leaf angle, heater response time and vegetation cover. Models project that the northeastern USA will warm by 2–3 °C by the end of the 21st century (Hayhoe *et al.* 2007) with summers experiencing up to a 10 % reduction in precipitation (Rawlins *et al.* 2012) and winter precipitation projected to increase by 11 to 14 % (Hayhoe *et al.* 2007).

**Figure 1.**
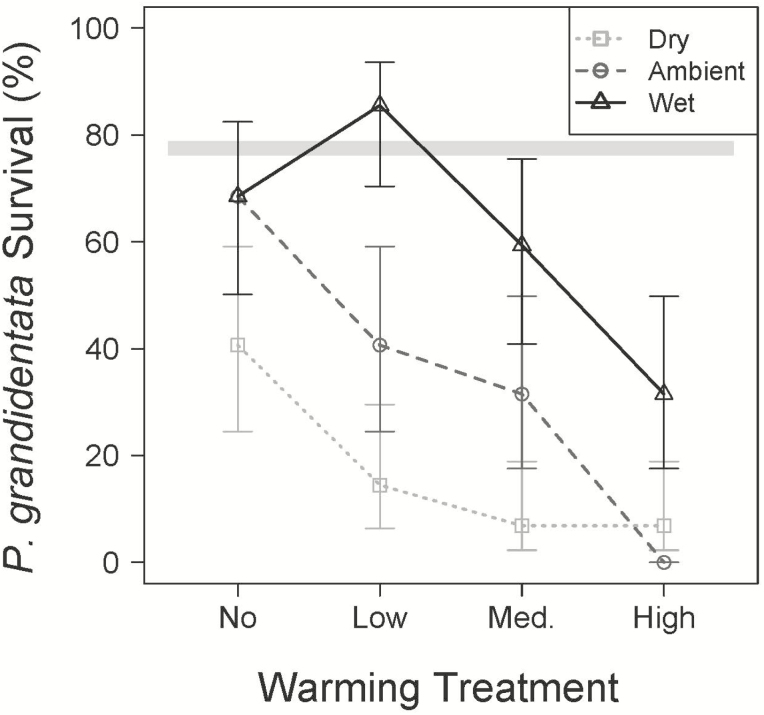
Probability of survival (±SE) from initial of planting (early spring) to sampling date (late July) for *P. grandidentata* in the wet (dark grey triangles), ambient (grey circles) and dry (light grey squares) precipitation treatments across the four warming treatments. The grey horizontal bar represents the mean survival (±SE) for the other five species combined.

Warming treatments were applied year-round using ceramic infrared heaters (e.g. Nexthermal FSR) mounted 1 m above the ground at each corner of every plot, facing towards the centre of the plot and downward at a 45° angle. The wattage varied by treatment: 200, 600 and 1000 W heaters for the low, medium and high warming treatments, respectively. Plant canopy temperatures of all ambient and high warming plots were monitored every 10 s by infrared radiometers (IRR-PN; Apogee Instruments, Logan, UT, USA). For each group of three warmed plots within a precipitation zone, power to all heaters was on a single circuit, and was continually adjusted based on the temperature difference between the unwarmed and high warming plots in that zone. The temperature control algorithms were designed to achieve a 4 °C difference between the unwarmed and high warming treatments when possible. Intermediate treatments received proportionally less warming because the heaters surrounding these plots had lower wattages [see **Supporting Information—Fig. 1]**. Hoeppner and Dukes (2012) reported that the heaters achieved target temperatures much of the time, but for periods during the day and in rainstorms temperatures often dipped below targets, however, a decrease in the diurnal temperature range, is similar to what is projected for the northeastern USA (Meehl *et al.* 2007).

Precipitation treatments were achieved using a roof of evenly spaced, clear polycarbonate slats that collected half of the precipitation over the dry treatments (year-round) and immediately delivered it to the wet treatments via an overhead sprinkler system (May–November). Soil moisture was fully recharged to water holding capacity during the winter months in all plots (Hoeppner and Dukes 2012). In order to adjust for the ~5 % reduction in photosynthetically active radiation (PAR) imposed by the polycarbonate slats, the ambient and wet sections of the greenhouse frames were covered by deer fencing, providing the same reduction in PAR.

Within each precipitation main plot, the four warming split-plots were arranged linearly from ambient to high, and spaced 1 m apart. In 2007, a 0.6 m deep trench was dug around each split-plot, and lined with polyethylene sheets to prevent lateral movement of water and nutrients between split-plots. All treatments were in effect as of July 2008. The herbaceous vegetation within and around the split-plots was clipped twice per growing season. Hoeppner and Dukes (2012) provide further details on the BACE experimental design.

Within each of the 36 split-plots, soil moisture was monitored weekly as relative extractable water (*θ*_R_) using pairs of time-domain reflectometry (TDR) waveguides installed vertically to provide integrated measures of volumetric soil moisture in the top 10 cm and top 30 cm; calculations in Vicca *et al.* (2012) and Smith *et al.* (2016). Soil temperature was monitored using custom-made linear temperature sensors placed at 2 and 10 cm below the soil surface. Measurements were recorded every 30 min throughout the year.

Within each of the 36 split-plots, four evenly spaced 0.5 m × 0.5 m subplots were cleared of grasses and forbs and designated for tree seedling planting. One seedling (<20 cm in height) of each of eight tree species was planted into each of these subplots in late April 2011, resulting in a total of 1152 seedlings planted. The eight tree species planted were: *A. rubrum* (red maple), *B. lenta* (sweet birch), *P. grandidentata* (big-toothed aspen), *P. serotina* (black cherry), *Q. rubra* (red oak), *U. americana* (American elm), *Betula populifolia* (grey birch) and *Pinus strobus* (white pine). Due to time and resource limitations *B. populifolia* and *P. strobus* were not measured in this study, leaving six species and 864 tree seedlings that were measured. Whole plant-level measurements (survival, TLA and herbivory) as well as leaf-level responses (SLA, LWC and foliar stoichiometry) were calculated to determine seedling responses to the treatments. All measurements were completed only over the 2011 growing season.

### Whole plant-level measurements

Seedling mortality counts were made by comparing the number of seedlings alive in late April as compared to late July. Dead seedlings were not included in the leaf-level analyses. In order to estimate total leaf production without destructively harvesting across the peak growing season, we used length and width measurements to develop species-specific leaf size categories (Kvet and Marshall 1971; Lu *et al.* 2004). Before the first measurements began, four distinct leaf size categories (undeveloped, small, medium and large) were established separately for each of the six species. The undeveloped category was used to represent the youngest leaves that had not yet reached the small stage. Ten representative leaves were chosen to represent each size category for each species. The length and width (to the nearest 0.1 cm) of each of these leaves were also measured and mean values were used to confirm visual categories during the sampling. The total number of leaves produced on each tree seedling and the size category for each leaf were determined for plant-level analyses in late July of 2011.

In late July of 2011, the magnitude of leaf-level insect herbivory was assessed as the percent of tissue removed using four categories for damage as previously determined by Chacon and Armesto (2006): 0 %, 1–15 %, 15–50 % and 50–100 %. The herbivory response variable was treated as continuous numbers 0, 1, 15 and 50 (meaning that 50 had 50 times more herbivory than 1). To ensure that visual estimates were consistent, the leaf size estimations and the herbivory levels were all completed by the same person (V.L.R.).

### Leaf-level measurements

After all plant-level analyses were completed, four green, representative leaves were collected from each of the tree seedlings for leaf-level analyses. When possible, the leaf samples were chosen to include at least one leaf from each of the small, medium and large size categories. For tree seedlings with limited leaf production, we took up to, but no more than, half of the leaves present. Each leaf collected was immediately weighed and scanned for leaf area using a CI-202 Portable Area Meter (CID Bio-Science, Camas, WA, USA). The mean leaf area for each size category of each species was calculated across all individuals. The estimated TLA for each individual tree seedling was then calculated as the number of leaves observed within each size category multiplied by the species-specific measured mean leaf area for each category, and summed across size categories.

Leaf samples were dried to a constant mass at 65 °C to determine dry weight and leaf-level water content (LWC) was calculated by subtracting dry weight from wet weight, and dividing by wet weight. Specific leaf area was measured as leaf area divided by dry weight. One small size category, dried leaf sample from each tree seedling was then ground and analysed for carbon and nitrogen concentration on a Costech ECS 4010 elemental analyzer (Costech Analytical Technologies Inc., Valencia, CA, USA).

### Data analysis and statistics

Seedling mortality, estimated TLA (m^2^), SLA (cm^2^ g^−1^), leaf herbivory (%), LWC (%), leaf tissue percent nitrogen by weight (%N) and leaf C:N ratio (g g^−1^) were analysed for all species together using linear mixed-effects models. Specific leaf area, LWC and herbivory were averaged for each individual tree. The mixed-effects models included a fully factorial combination of precipitation treatment, warming treatment and species as fixed effects. Block, precipitation treatment within block and warming treatment within precipitation treatment within block were included as random effects in each model to represent the experimental design of BACE. For model fitting, we used the ‘lmer’ function from the *lme4* package (Bates *et al.* 2015) in the statistical programming environment R (R Core Development Team 2015). As seedling mortality was binomial (died or survived), we used a logit-link binomial generalized linear model, implemented with function ‘glmer’ in *lme4*. Model convergence could not be achieved for the mortality model with all species included, due to the low mortality of some species. Therefore, a separate seedling mortality model was run for each species with >15 % total mortality (*A. rubrum*, *P. grandidentata* and *P. serotina*). To evaluate the level of significance for each fixed effect, we calculated a Wald χ^2^ statistic and performed a Type-II Wald test using the ‘ANOVA’ function in the ‘car’ package (Fox and Weisberg 2011) in R. *Post hoc* comparison of means was done using Tukey’s least squared difference tests utilizing the ‘lsmeans’ package (Lenth 2016) in R.

## Results

### Treatment effects on seedling mortality

Over the course of this experiment and throughout all plots *B. lenta*, *U. americana* and *Q. rubra* had low (<13.2 %) tree mortality; only 7, 8 and 19, respectively, out of 144 each. *Prunus serotina* and *A. rubrum* had moderate levels of mortality (43.1 and 45.8 %) with 82 and 78 of each species surviving. The highest mortality rate (61.9 %) was in *P. grandidentata*; only 55 out of the 144 seedlings survived. Among the species with >15 % mortality, only *P. grandidentata* showed a significant response to the treatments. Both warming and reduced precipitation caused greater *P. grandidentata* mortality (Fig. 1).

### Total leaf area

**Figure 2.**
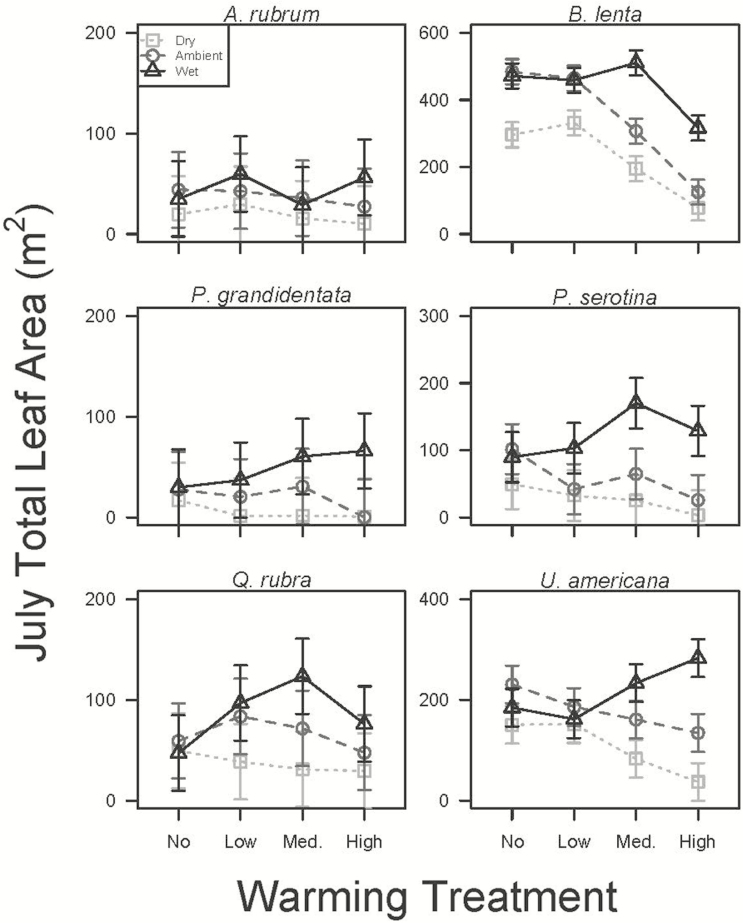
Estimated TLA in late July (mean ± SE) for *A. rubrum*, *B. lenta*, *P. grandidentata*, *P. serotina*, *Q. rubra* and *U. americana* in the wet (dark grey triangles), ambient (grey circles) and dry (light grey squares) precipitation treatments across the four warming treatments.

Total leaf area was reduced by 83 % under the combination of reduced precipitation and warming as compared to controls (warming by precipitation interaction; *P* < 0.001; see Supporting Information—Fig. S2). There were also significant species by warming (*P* < 0.05; Table 1) and species by precipitation (*P* < 0.05; Table 1) interactions. *Post hoc* analyses revealed that TLA was 49 and 51 % lower in reduced precipitation compared to added precipitation for *B. lenta* and *U. americana*, respectively (both *P* < 0.05; Fig. 2; [see **Supporting Information—Table S1**), but precipitation did not have an effect on other species (precipitation by species: *P* < 0.05). Total leaf area was 19 and 58% lower in medium and high warming, respectively, compared to no warming in *B. lenta*, but warming did not have a direct effect in other species (warming by species: *P* < 0.05; Fig. 2; [see **Supporting Information—Table S2**).

**Table 1.** Mixed model results for estimated TLA, leaf herbivory damage, SLA, LWC, leaf carbon (%C), leaf nitrogen (%N) and leaf C:N. *P*-values < 0.05 and 0.10 are bolded and italicized, respectively. Df, degrees of freedom; χ^2^, Wald’s chi-squared statistic.

	Df	TLA		Herbivory		SLA		LWC		Leaf C		Leaf N		Leaf C:N	
		χ^2^	*P*-value	χ^2^	*P*-value	χ^2^	*P*-value	χ^2^	*P*-value	χ^2^	*P*-value	χ^2^	*P*-value	χ^2^	*P*-value
Precipitation (P)	2	8.96	**0.011**	2.31	0.314	2.39	0.303	3.91	0.141	0.80	0.671	10.91	**0.004**	22.68	**<0.001**
Warming (W)	3	23.78	**<0.001**	4.40	0.221	0.83	0.842	2.41	0.491	3.36	0.340	3.16	0.368	5.30	0.151
Species (S)	5	993.15	**<0.001**	207.19	**<0.001**	238.98	**<0.001**	89.00	**<0.001**	224.96	**<0.001**	184.79	**<0.001**	212.22	**<0.001**
P × W	6	21.84	**0.001**	14.08	**0.029**	5.15	0.524	6.98	0.323	8.38	0.212	2.30	0.890	3.70	0.717
P × S	10	57.73	**<0.001**	44.59	**<0.001**	17.75	***0.059***	40.18	**<0.001**	19.36	**0.036**	43.19	**<0.001**	38.28	**<0.001**
W × S	15	110.11	**<0.001**	38.88	**0.001**	10.25	0.804	29.98	**0.012**	10.82	0.765	25.63	**0.042**	31.75	**0.007**
P × W × S	30	30.60	0.435	153.60	**<0.001**	34.22	0.272	41.43	***0.080***	39.93	0.106	44.23	**0.045**	15.35	***0.988***

### Insect herbivory

**Figure 3.**
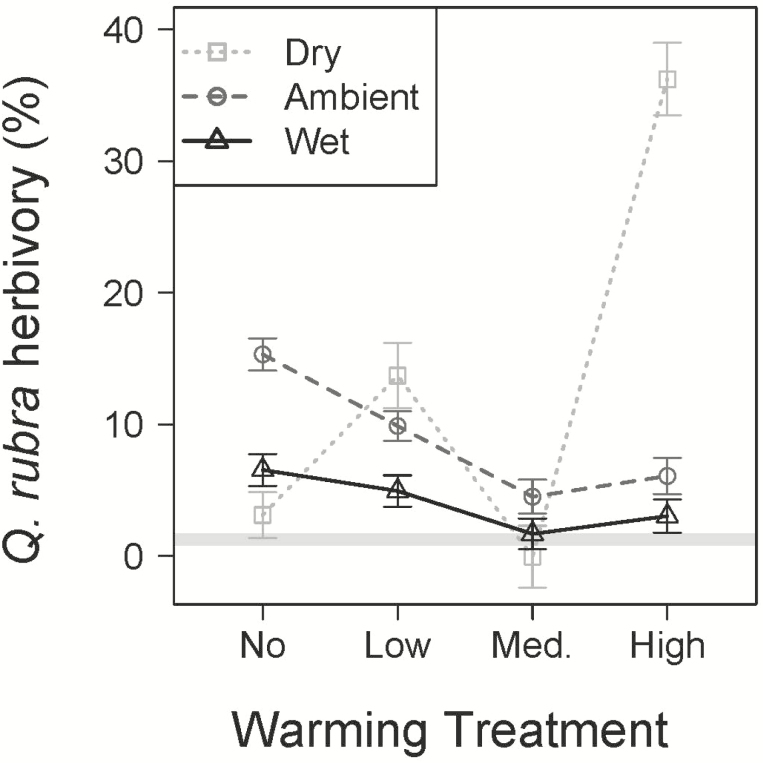
Mean percent leaf level insect herbivory (±SE) for *Q. rubra* in the wet (dark grey triangles), ambient (grey circles) and dry (light grey squares) precipitation treatments across the four warming treatments measured in late July. The grey horizontal bar represents the mean percent herbivory for the other five species combined.

A significant three-way interaction between species, warming treatment and precipitation treatment (*P* < 0.05; Table 1) indicated that *Q. rubra* was unique from the rest of the species (Fig. 3). *Quercus rubra* experienced over four times more herbivory damage than the species with the second highest rates, *A. rubrum* (*P* < 0.05; [see **Supporting Information—Fig. S3**). Rates of herbivory on *Q. rubra* were greatest in the combined reduced precipitation, high warming plots, showing an increase of 137 % as compared to the ambient precipitation, no warming plots (*P* < 0.05; Fig. 3).

### SLA and LWC

Specific leaf area varied by species, with the highest values seen in *B. lenta*, followed by *P. serotina*, *A. rubrum*, *U. americana*, *P. grandidentata* and *Q. rubra* [see **Supporting Information—Table S3**]. However, SLA did not respond to the warming or precipitation treatments in any of these species (*P* > 0.05 for all main effects and interactions; Table 1; see **Supporting Information—Fig. S4**).

In general, LWC was relatively unaffected by the treatments. However, dry treatments reduced LWC of *Q. rubra* by 35 % (*P* < 0.05; [see **Supporting Information—Table S1; Fig. S5**). In addition, the highest level of warming reduced the LWC of *B. lenta* by 21 % as compared to the unwarmed plots (*P* < 0.05; [see **Supporting Information—Table S2; Fig. S5**).

### Foliar stoichiometry

**Figure 4.**
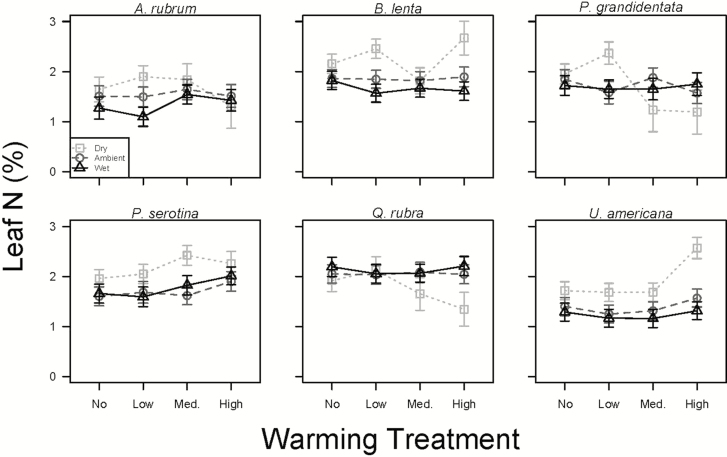
Foliar nitrogen by weight (percent; mean ± SE) of *A. rubrum*, *B. lenta*, *P. grandidentata*, *P. serotina*, *Q. rubra* and *U. americana* in the wet (dark triangles), ambient (grey circles) and dry (light squares) precipitation treatments across the four warming treatments measured in late July.

**Figure 5.**
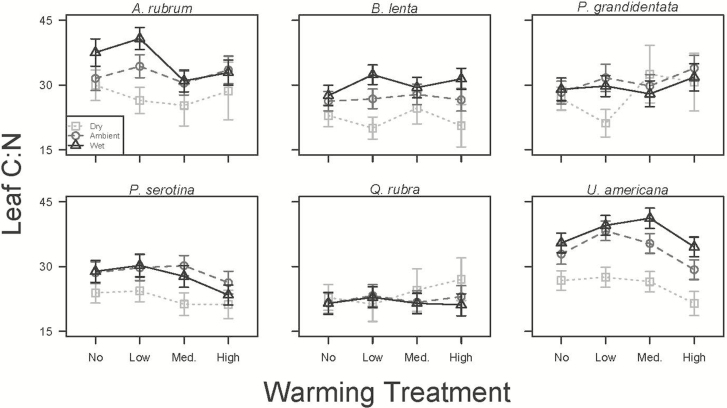
Leaf carbon:nitrogen ratio (±SE) for *A. rubrum*, *B. lenta*, *P. grandidentata*, *P. serotina*, *Q. rubra* and *U. americana* in the wet (dark grey triangles), ambient (grey circles) and dry (light grey squares) precipitation treatments across the four warming treatments measured in late July.

Foliar percent N responded inconsistently to the climate treatments, with only *U. americana* increasing leaf N by 84 % in the driest and warmest plots compared to plots with reduced precipitation (*P* < 0.05; Table 1; Fig. 4). Foliar carbon content was relatively stable across the treatments (Table 1), except for a large increase in the high warmed, dry treatments for *B. lenta* [see **Supporting Information—Fig. S6]**. Four of the six species, *P. serotina*, *B. lenta*, *A. rubrum* and *U. americana*, had higher C:N in plots receiving greater precipitation (*P* < 0.05; Fig. 5), while *Q. rubra* and *P. grandidentata* had no change with the precipitation treatment (*P* > 0.05; Fig. 5; see **Supporting Information—Table S1**). *Ulmus americana* was also the only species to respond significantly (*P* < 0.05; Fig. 5) to warming, with the high heating reducing leaf C:N by 10 % as compared to the unwarmed plots [see **Supporting Information—Table S2**].

## Discussion

Seedling mortality of *P. grandidentata* increased in warmer and drier conditions, and this species showed limited plasticity in leaf-level responses to the manipulations. The combination of warming and reduced precipitation suppressed TLA across species, and strongly in the most productive species, *B. lenta* and *U. americana*. Herbivory primarily affected *Q. rubra*, with the highest rates occurring in the warmest, driest plots. At the leaf level, SLA and foliar N were rarely affected by the treatments, while leaf C:N increased in wetter plots, most notably in *B. lenta*, *A. rubrum*, *P. serotina* and *U. americana*.

**Figure 6.**
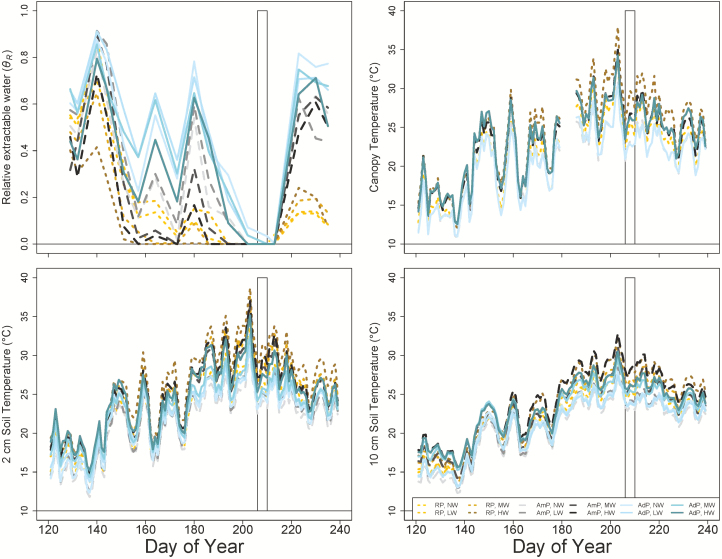
Mean relative extractable water (*θ*_R_; unitless; top left), canopy temperature (top right), soil temperature at 2 cm depth (bottom left), soil temperature at 10 cm depth (bottom right) in the added precipitation (AdP; blue, solid lines), ambient precipitation (AmP; grey and black, dashed lines) and reduced precipitation (brown, dotted lines) over the course of the experiment. Darker colours within each precipitation treatment indicate higher levels of warming (NW, no warming; LW, low warming; MW, medium warming; HW, high warming). Means are for each plot type during each measurement date (*n* = 3). White box indicates the plant measurement dates.

Consistent with our expectations, the warming treatments combined with either ambient or reduced precipitation progressively reduced estimated TLA of most species, likely as a response to whole-plant water stress induced by warming-driven increases in water demand (i.e. vapour pressure deficit) and reductions in water supply (i.e. soil moisture) (Smith *et al.*, 2016). This was likely to be especially pronounced in our study based upon the warm and dry background conditions prior to sampling date (Fig. 6). Using four of the same tree species, and also at BACE, Smith *et al.* (2016) found that net photosynthesis and transpiration rates decreased with warming as a consequence of the indirect effect of the warming treatments on soil moisture. Our results highlight two possible strategies for reducing whole-plant water loss in combined warm, dry conditions: reduced growth or the shedding of leaves. That the TLA response was greatest in the warmest, driest plots, but the physiological responses occurred in response to warming and drying alone (Smith *et al.*, 2016) suggesting that leaf area reduction later in the growing season could potentially be a secondary response to drought in these species. The interactions of temperature with the reduced precipitation treatment on insect damage in *Q. rubra* were particularly complex and the fluctuations between no, low and medium warming are difficult to explain biologically (Fig. 3). However, the combined high temperature, dry treatment dramatically increased herbivory damage (Fig. 3), suggesting that leaves grown in hot, dry conditions are more favourable for oak herbivores. Although the effects of temperature on insect performance differ greatly in different habitats, host plants, insect life history strategies and complex trophic interactions, there is much data to suggest that temperature itself enhances localized insect herbivores through survival, abundance and development (review in Bale et al., 2002). Interestingly, Carnicer *et al.* (2011) found trends of increased crown defoliation in southern European forests after periods of severe drought, which they suggest is due to different species-specific responses to increased water deficit pressures within complex food webs.

As we did not measure what types of insect herbivores were consuming the leaves, we cannot be sure whether they were attracted to the leaves or present in higher numbers within the high temperature, dry plots. In a study of *Q. rubra* leaf metabolite responses at BACE, Suseela *et al.* (2015) found that both warmed and drier conditions increased the concentration of soluble proteins in green leaf tissue by over 75 % as compared to controls. Leaves in the dry, high warming treatment of that study also had a markedly different metabolite profile and the highest tannin concentrations. The increased damage we observed could suggest that some herbivores may be able to detect the enhanced nutritional quality of these climate-stressed plants and tolerate the higher tannin content—but could also suggest greater consumption by the herbivore individuals that were present.

Under favourable conditions (e.g. warm and wet), plants tend to produce higher SLA (Poorter *et al.* 2009). However, we found little consistent response of SLA to the climate treatment manipulations we imposed (Table 1). This suggests that, in these deciduous species, the primary means of response to climatic change is not through alterations of leaf morphology, but through a change in canopy properties, namely leaf area. In this experiment, the individuals were planted in each plot before leaf onset in the spring, but had not been acclimated to the treatments over multiple growing seasons. As such, our results suggest that, within a single growing season, leaf morphology is generally less plastic than TLA.

We expected foliar N to increase (and foliar C:N to decrease) with warming and added precipitation. However, the treatments had no consistent effect on foliar percent N (Table 1). *Ulmus americana* had the overall lowest foliar N level, and was the only species to respond to the treatments, with greater leaf N concentrations in the warmer and drier plots. Auyeung (2012) found the availability of summer soil ammonium and nitrate to be highest in the dry treatment at BACE. Other work at BACE found that warming increased foliar N in herbaceous plant tissue (Rodgers *et al.* 2012) but had little effect on soil N cycling rates (Auyeung *et al*. 2013).

While we did not see a strong response of leaf N to the treatments (Fig. 4), leaf C:N tended to decrease in drier plots in all species except *P. grandidentata* and *Q. rubra* (Fig. 5), indicating a reduction in foliar C in dry conditions (Table 1). Combined with a reduction in TLA in these plots, this result suggests that seedlings grown under dry conditions are likely to take up and store less carbon, at least in their leaves. Interestingly, the two species that did not show C:N changes, *P. grandidentata* and *Q. rubra*, showed unique whole-plant increases in mortality and herbivory, respectively, in response to warm, dry conditions.

We had hypothesized that *B. lenta* and *P. grandidentata* would be more negatively affected by the warmed and dry treatments than the other species because their climatic niches and range sizes are smaller, and their ranges lie farther north and east. This hypothesis was supported, but in somewhat different ways for each species. Our results confirm the low drought tolerance of *P. grandidentata* with large percentages of these seedlings dying in both the dry and warmed conditions (Fig. 1), whereas other species’ seedlings had much higher survivorship. Although *B. lenta* seedlings survived environmental conditions in all of the treatments, drier conditions reduced leaf production of *B. lenta* (along with *U. americana*) more strongly than the other species, and *B. lenta* TLA was most strongly suppressed by warming (Fig. 2). This suppression of TLA could be a consequence of limited C inputs; working with seedlings of four of the same tree species at BACE, Smith *et al.* (2016) found that the leaf gas exchange rates of *B. lenta* were more sensitive to soil moisture than the gas exchange rates of *P. serotina*, *Q. rubra* or *U. americana*. Taking these results together with the trend towards increasing leaf N content [see **Supporting Information—Table S2]**, *B. lenta* appears to be more conservative when stressed and could be considered one of the more isohydric species we studied. The USDA plants database classifies *P. grandidentata* and *Q. rubra* as having low tolerance to drought, while *A. rubrum*, *B. lenta*, *P. serotina* and *U. americana* are listed as having a medium level (USDA 2017). The two low-tolerance species each had unique responses to the driest treatments in this study, with high seedling mortality in *P. grandidentata* (Fig. 1) and high herbivory in *Q. rubra* (Fig. 3). However, responses of growth (measured at TLA) and other leaf properties did not clearly distinguish these species from the other four (Fig. 2). We would not expect our measurements to perfectly classify species’ drought tolerances, as we measured these trees only as seedlings, although the seedling stage may be the most critical for determining long-term persistence of a tree species.

Our findings that plants generally responded more strongly to the reduced precipitation treatment than to warming, and that warming and reduced precipitation together had the strongest effects (Table 1; see **Supporting Information—Fig. S2**), parallel those of other studies from the BACE site (Tharayil *et al.* 2011; Hoeppner and Dukes 2012; Rodgers *et al.* 2012; Suseela *et al.* 2012; Suseela *et al.* 2015; Smith *et al.* 2016). Interestingly, there is little evidence of added precipitation ameliorating any negative responses to the high warming treatment. Taken together, these studies suggest that future projected warming conditions in the northeastern USA (Hayhoe *et al.* 2007) in combination with extended dry periods over the growing season (Rawlins *et al.* 2012) may result in substantial shifts in species composition, declines in whole-plant growth and altered leaf-level health.

## Conclusions

Warm and dry conditions suppressed seedling growth in these six common New England tree species, as measured by TLA. These conditions also increased mortality and herbivory of *P. grandidentata* and *Q. rubra*, respectively. For the other four species, warm, dry conditions decreased foliar C and C:N. This study indicates that climatic warming is likely to increase the sensitivity of carbon uptake to precipitation in young trees. Dry years in a warming climate are likely to increasingly suppress production through species-specific combinations of mortality, herbivory, reduced leaf area and reduced per-leaf C uptake.

## Sources of Funding

The Boston-Area Climate Experiment was constructed with funding from the National Science Foundation (grant # DEB-0546670), with further support from NSF (DEB-1146279) and the U.S. Department of Energy’s Office of Science (BER), through the Terrestrial Ecosystem Science programme and the Northeastern Regional Center of the National Institute for Climatic Change Research. Faculty and student summer research funds were provided by the Babson Faculty Research Fund. Partial support for J.S.D.’s participation in this project was provided by the USDA’s National Institute of Food and Agriculture, through Hatch project 1000026 and AFRI grant 2015-67003-23485. This is paper no. 1821 of the Purdue Climate Change Research Center (PCCRC).

## Contributions by the Authors

V.L.R., S.S.H. and J.S.D. conceived of the project. V.L.R., N.G.S. and S.S.H. took the field measurements. V.L.R. prepped lab samples. N.G.S. and S.S.H. analysed the data. All authors contributed to the writing of the article.

## Conflict of Interest

None declared.

## Supporting Information

The following additional information is available in the online version of this article—

**Table S1.** Results from *post hoc* Tukey’s tests for TLA, LWC and C:N across precipitation treatments. Key: SE, standard error of the least square means; Ddf, denominator degrees of freedom; lowerCL, lower confidence limit; upperCL, upper confidence limit; Group, indicates statistically different mean values for treatment within species based on Tukey’s HSD (α = 0.05). Ambient denotes treatments receiving ambient precipitation.

**Table S2.** Results from *post hoc* Tukey’s tests for TLA, LWC and C:N across warming treatments. Key: SE, standard error of the least square means; Ddf, denominator degrees of freedom; lowerCL, lower confidence limit; upperCL, upper confidence limit; Group, indicates statistically different mean values for treatment within species based on Tukey’s HSD (α = 0.05). Control denotes the unwarmed treatments.

**Table S3.** Specific leaf area (SLA) by species (mean ± SE) with different lower case letters indicating significant differences (*P* < 0.05) from a Tukey’s HSD test.

**Figure S1.** Hourly canopy temperature averaged across plot type for DOY 121-239 in the added precipitation (AdP; blue, solid lines), ambient precipitation (AmP; grey and black, dashed lines) and reduced precipitation (brown, dotted lines) over the course of the experiment. Darker colours within each precipitation treatment indicate higher levels of warming (NW, no warming; HW, high warming). Means are for the three plot types over 119 days (*n* = 357).

**Figure S2.** Estimated total leaf (surface) area in late July (±SE) for all species averaged together in the wet (dark grey triangles), ambient (grey circles) and dry (light grey squares) precipitation treatments across the four warming treatments.

**Figure S3.** Leaf level insect herbivory (percent; mean ± SE) for *A. rubrum*, *B. lenta*, *P. grandidentata*, *P. serotina*, *Q. rubra* and *U. americana* in the wet (dark triangles), ambient (grey circles) and dry (light squares) precipitation treatments across the four warming treatments measured in late July.

**Figure S4.** Specific leaf area (mean ± SE) for *A. rubrum*, *B. lenta*, *P. grandidentata*, *P. serotina*, *Q. rubra* and *U. americana* in the wet (dark triangles), ambient (grey circles) and dry (light squares) precipitation treatments across the four warming treatments measured in late July.

**Figure S5.** Leaf-level water content (percent; mean ± SE) of *A. rubrum*, *B. lenta*, *P. grandidentata*, *P. serotina*, *Q. rubra* and *U. americana* in the wet (dark triangles), ambient (grey circles) and dry (light squares) precipitation treatments across the four warming treatments measured in late July.

**Figure S6.** Foliar carbon by weight (percent; mean ± SE) of *A. rubrum*, *B. lenta*, *P. grandidentata*, *P. serotina*, *Q. rubra* and *U. americana* in the wet (dark triangles), ambient (grey circles) and dry (light squares) precipitation treatments across the four warming treatments measured in late July.

Supporting Information
