# Supplementary material for: Warming increases the sensitivity of seedling growth capacity to rainfall in six temperate deciduous tree species
Source: AoB Plants. 2018 Jan 17;10(1):ply003. doi: 10.1093/aobpla/ply003 (PMC5815139; doi:10.1093/aobpla/ply003)

**Supporting Information Table 1.** Results from post-hoc tukey’s tests for TLA, LWC and CN across precipitation treatments. Key: SE, standard error of the least square means; Ddf, denominator degrees of freedom; lowerCL, lower confidence limit; upperCL, upper confidence limit; Group, indicates statistically different mean values for treatment within species based on Tukey’s HSD (alpha =0.05). Ambient denotes treatments receiving ambient precipitation.

|  |  |  |  | **TLA** |  |  |  |  |  |  | **LWC** |  |  |  |  |  |  | **C:N** |  |  |  |
| --- | --- | --- | --- | --- | --- | --- | --- | --- | --- | --- | --- | --- | --- | --- | --- | --- | --- | --- | --- | --- | --- |
| **Species** | **Precipitation** | **Estimate** | **SE** | **Ddf** | **LowerCL** | **UpperCL** | **Group** |  | **Estimate** | **SE** | **Ddf** | **LowerCL** | **UpperCL** | **Group** |  | **Estimate** | **SE** | **Ddf** | **LowerCL** | **UpperCL** | **Group** |
| ***A. rubrum*** | **Dry** | 18.756 | 26.439 | 10.472 | -39.795 | 77.307 | a |  | 36.232 | 4.245 | 21.887 | 27.426 | 45.038 | a |  | 27.570 | 2.491 | 97.533 | 22.626 | 32.513 | a |
|  | **Ambient** | 37.387 | 26.439 | 10.472 | -21.164 | 95.938 | a |  | 49.767 | 3.369 | 9.006 | 42.147 | 57.388 | a |  | 32.464 | 1.611 | 22.461 | 29.127 | 35.800 | ab |
|  | **Wet** | 44.932 | 26.439 | 10.472 | -13.619 | 103.483 | a |  | 45.244 | 3.346 | 8.760 | 37.644 | 52.844 | a |  | 35.537 | 1.586 | 21.229 | 32.240 | 38.834 | b |
| ***B. lenta*** | **Dry** | 225.143 | 26.439 | 10.472 | 166.592 | 283.694 | a |  | 44.076 | 3.730 | 13.175 | 36.029 | 52.124 | a |  | 22.061 | 1.951 | 40.166 | 18.117 | 26.004 | a |
|  | **Ambient** | 345.119 | 26.439 | 10.472 | 286.568 | 403.670 | b |  | 49.625 | 3.205 | 7.383 | 42.124 | 57.125 | a |  | 26.878 | 1.407 | 13.201 | 23.843 | 29.913 | ab |
|  | **Wet** | 439.304 | 26.439 | 10.472 | 380.753 | 497.855 | b |  | 48.793 | 3.191 | 7.254 | 41.300 | 56.286 | a |  | 30.210 | 1.392 | 12.642 | 27.194 | 33.227 | b |
| ***P. grandidentata*** | **Dry** | 5.100 | 26.439 | 10.472 | -53.451 | 63.651 | a |  | 43.103 | 4.492 | 27.324 | 33.891 | 52.315 | a |  | 27.815 | 2.692 | 129.338 | 22.489 | 33.142 | a |
|  | **Ambient** | 19.631 | 26.439 | 10.472 | -38.920 | 78.182 | a |  | 44.468 | 3.434 | 9.722 | 36.786 | 52.150 | a |  | 30.917 | 1.608 | 22.402 | 27.585 | 34.249 | a |
|  | **Wet** | 48.279 | 26.439 | 10.472 | -10.272 | 106.830 | a |  | 39.325 | 3.353 | 8.829 | 31.719 | 46.932 | a |  | 29.647 | 1.594 | 21.515 | 26.337 | 32.957 | a |
| ***P. serotina*** | **Dry** | 27.474 | 26.439 | 10.472 | -31.077 | 86.025 | a |  | 45.336 | 3.346 | 8.707 | 37.728 | 52.945 | a |  | 22.701 | 1.548 | 18.850 | 19.460 | 25.942 | a |
|  | **Ambient** | 58.475 | 26.439 | 10.472 | -0.076 | 117.026 | a |  | 56.942 | 3.291 | 8.198 | 49.385 | 64.499 | a |  | 28.712 | 1.516 | 17.609 | 25.522 | 31.903 | b |
|  | **Wet** | 122.933 | 26.439 | 10.472 | 64.382 | 181.484 | a |  | 53.873 | 3.241 | 7.715 | 46.350 | 61.395 | a |  | 27.582 | 1.456 | 15.051 | 24.479 | 30.684 | ab |
| ***Q. rubra*** | **Dry** | 37.084 | 26.439 | 10.472 | -21.467 | 95.635 | a |  | 32.242 | 4.045 | 18.097 | 23.746 | 40.737 | a |  | 23.945 | 2.280 | 70.420 | 19.398 | 28.492 | a |
|  | **Ambient** | 65.527 | 26.439 | 10.472 | 6.976 | 124.078 | a |  | 49.283 | 3.251 | 7.815 | 41.755 | 56.812 | b |  | 22.395 | 1.458 | 15.180 | 19.291 | 25.499 | a |
|  | **Wet** | 85.910 | 26.439 | 10.472 | 27.359 | 144.461 | a |  | 43.552 | 3.227 | 7.587 | 36.038 | 51.065 | ab |  | 21.762 | 1.439 | 14.371 | 18.684 | 24.840 | a |
| ***U. americana*** | **Dry** | 105.644 | 26.439 | 10.472 | 47.093 | 164.195 | a |  | 45.811 | 3.254 | 7.783 | 38.270 | 53.353 | a |  | 25.575 | 1.430 | 13.716 | 22.502 | 28.648 | a |
|  | **Ambient** | 178.067 | 26.439 | 10.472 | 119.516 | 236.618 | ab |  | 53.159 | 3.173 | 7.090 | 45.675 | 60.643 | a |  | 33.951 | 1.368 | 11.811 | 30.964 | 36.937 | b |
|  | **Wet** | 215.728 | 26.439 | 10.472 | 157.177 | 274.279 | b |  | 43.517 | 3.168 | 7.041 | 36.035 | 50.998 | a |  | 37.695 | 1.368 | 11.812 | 34.709 | 40.682 | b |

**Supporting Information Table 2.** Results from post-hoc tukey’s tests for TLA, LWC and CN across warming treatments. Key: SE, standard error of the least square means; Ddf, denominator degrees of freedom; lowerCL, lower confidence limit; upperCL, upper confidence limit; Group, indicates statistically different mean values for treatment within species based on Tukey’s HSD (alpha =0.05). Control denotes the unwarmed treatments

|  |  |  |  | **TLA** |  |  |  |  |  |  | **LWC** |  |  |  |  |  |  | **C:N** |  |  |  |
| --- | --- | --- | --- | --- | --- | --- | --- | --- | --- | --- | --- | --- | --- | --- | --- | --- | --- | --- | --- | --- | --- |
| **Species** | **Warming** | **Estimate** | **SE** | **Ddf** | **LowerCL** | **UpperCL** | **Group** |  | **Estimate** | **SE** | **Ddf** | **LowerCL** | **UpperCL** | **Group** |  | **Estimate** | **SE** | **Ddf** | **LowerCL** | **UpperCL** | **Group** |
| ***A. rubrum*** | **Control** | 32.918 | 22.693 | 10.849 | -17.113 | 82.950 | a |  | 47.685 | 3.088 | 46.961 | 41.472 | 53.898 | a |  | 33.013 | 1.835 | 169.504 | 29.390 | 36.635 | a |
|  | **Low** | 43.952 | 22.693 | 10.849 | -6.079 | 93.984 | a |  | 41.341 | 2.831 | 33.474 | 35.585 | 47.097 | a |  | 33.849 | 1.575 | 103.151 | 30.725 | 36.973 | a |
|  | **Medium** | 26.688 | 22.693 | 10.849 | -23.343 | 76.720 | a |  | 40.637 | 3.284 | 59.174 | 34.065 | 47.209 | a |  | 28.904 | 2.020 | 223.105 | 24.923 | 32.886 | a |
|  | **High** | 31.208 | 22.693 | 10.849 | -18.823 | 81.239 | a |  | 45.328 | 4.066 | 113.592 | 37.273 | 53.383 | a |  | 31.661 | 2.660 | 338.800 | 26.429 | 36.892 | a |
| ***B. lenta*** | **Control** | 417.206 | 22.693 | 10.849 | 367.175 | 467.237 | a |  | 52.239 | 2.640 | 25.587 | 46.809 | 57.670 | a |  | 25.592 | 1.382 | 66.696 | 22.835 | 28.350 | a |
|  | **Low** | 418.219 | 22.693 | 10.849 | 368.187 | 468.250 | a |  | 48.576 | 2.631 | 25.203 | 43.160 | 53.991 | ab |  | 26.424 | 1.369 | 64.292 | 23.689 | 29.160 | a |
|  | **Medium** | 337.507 | 22.693 | 10.849 | 287.476 | 387.538 | b |  | 48.074 | 2.934 | 37.776 | 42.132 | 54.016 | ab |  | 27.316 | 1.654 | 104.439 | 24.036 | 30.597 | a |
|  | **High** | 173.156 | 22.693 | 10.849 | 123.124 | 223.187 | c |  | 41.103 | 3.377 | 58.223 | 34.343 | 47.863 | b |  | 26.199 | 2.029 | 180.583 | 22.195 | 30.202 | a |
| ***P. grandidentata*** | **Control** | 24.713 | 22.693 | 10.849 | -25.318 | 74.744 | a |  | 43.570 | 2.771 | 30.965 | 37.918 | 49.221 | a |  | 28.048 | 1.522 | 95.302 | 25.026 | 31.071 | a |
|  | **Low** | 19.517 | 22.693 | 10.849 | -30.515 | 69.548 | a |  | 41.248 | 3.025 | 43.344 | 35.149 | 47.347 | a |  | 27.541 | 1.697 | 139.354 | 24.186 | 30.896 | a |
|  | **Medium** | 30.879 | 22.693 | 10.849 | -19.152 | 80.911 | a |  | 44.122 | 3.951 | 112.933 | 36.294 | 51.949 | a |  | 30.105 | 2.585 | 344.323 | 25.020 | 35.190 | a |
|  | **High** | 22.238 | 22.693 | 10.849 | -27.793 | 72.269 | a |  | 40.256 | 4.142 | 121.093 | 32.055 | 48.456 | a |  | 32.144 | 2.654 | 341.603 | 26.924 | 37.365 | a |
| ***P. serotina*** | **Control** | 80.288 | 22.693 | 10.849 | 30.256 | 130.319 | a |  | 54.664 | 2.680 | 27.194 | 49.166 | 60.162 | a |  | 27.139 | 1.427 | 75.511 | 24.297 | 29.980 | a |
|  | **Low** | 59.121 | 22.693 | 10.849 | 9.090 | 109.152 | a |  | 54.134 | 2.823 | 33.250 | 48.393 | 59.875 | a |  | 28.123 | 1.573 | 104.762 | 25.004 | 31.243 | a |
|  | **Medium** | 86.601 | 22.693 | 10.849 | 36.570 | 136.632 | a |  | 52.071 | 2.696 | 27.846 | 46.547 | 57.596 | a |  | 26.426 | 1.445 | 79.807 | 23.551 | 29.302 | a |
|  | **High** | 52.500 | 22.693 | 10.849 | 2.469 | 102.531 | a |  | 47.333 | 2.911 | 33.683 | 41.414 | 53.252 | a |  | 23.639 | 1.597 | 94.578 | 20.469 | 26.809 | a |
| ***Q. rubra*** | **Control** | 51.886 | 22.693 | 10.849 | 1.855 | 101.917 | a |  | 45.728 | 2.773 | 31.000 | 40.071 | 51.384 | a |  | 21.961 | 1.521 | 92.433 | 18.941 | 24.982 | a |
|  | **Low** | 73.069 | 22.693 | 10.849 | 23.038 | 123.100 | a |  | 41.678 | 3.021 | 43.112 | 35.586 | 47.771 | a |  | 22.487 | 1.768 | 152.198 | 18.994 | 25.980 | a |
|  | **Medium** | 75.389 | 22.693 | 10.849 | 25.358 | 125.421 | a |  | 36.045 | 3.294 | 58.806 | 29.453 | 42.637 | a |  | 22.599 | 2.007 | 195.364 | 18.640 | 26.558 | a |
|  | **High** | 51.018 | 22.693 | 10.849 | 0.986 | 101.049 | a |  | 43.318 | 3.388 | 58.985 | 36.538 | 50.099 | a |  | 23.755 | 2.028 | 179.360 | 19.754 | 27.756 | a |
| ***U. americana*** | **Control** | 188.695 | 22.693 | 10.849 | 138.664 | 238.727 | a |  | 46.881 | 2.589 | 23.745 | 41.534 | 52.228 | a |  | 31.717 | 1.329 | 58.709 | 29.059 | 34.376 | ab |
|  | **Low** | 166.599 | 22.693 | 10.849 | 116.567 | 216.630 | a |  | 47.628 | 2.590 | 23.756 | 42.281 | 52.976 | a |  | 35.123 | 1.329 | 58.679 | 32.464 | 37.782 | a |
|  | **Medium** | 159.141 | 22.693 | 10.849 | 109.110 | 209.172 | a |  | 48.287 | 2.590 | 23.759 | 42.939 | 53.635 | a |  | 34.353 | 1.342 | 60.862 | 31.669 | 37.037 | a |
|  | **High** | 151.482 | 22.693 | 10.849 | 101.451 | 201.513 | a |  | 47.186 | 2.739 | 26.541 | 41.562 | 52.810 | a |  | 28.435 | 1.414 | 61.530 | 25.608 | 31.262 | b |

**Supporting Information Table 3.** Specific leaf area (SLA) by species (mean ± standard error) with different lower case letters indicating significant differences (*P* < 0.05) from a Tukey’s HSD test.

| **Species** | **SLA (cm2 g-1)** |
| --- | --- |
| *B. lenta* | 212.23 ± 5.77 ^a^ |
| *P. serotina* | 189.71 ± 5.42 ^b^ |
| *A. rubrum* | 175.92 ± 7.11 ^b^ |
| *U. americana* | 154.26 ± 4.94 ^c^ |
| *P. grandidentata* | 142.16 ± 7.51 ^cd^ |
| *Q. rubra* | 134.06 ± 6.42 ^d^ |

**Figure S1.** Hourly canopy temperature averaged across plot type for DOY 121-239 in the added precipitation (AdP; blue, solid lines), ambient precipitation (AmP; grey and black, dashed lines), and reduced precipitation (brown, dotted lines) over the course of the experiment. Darker colors within each precipitation treatment indicate higher levels of warming (NW, no warming; HW, high warming). Means are for the 3 plot types over 119 days (n = 357).


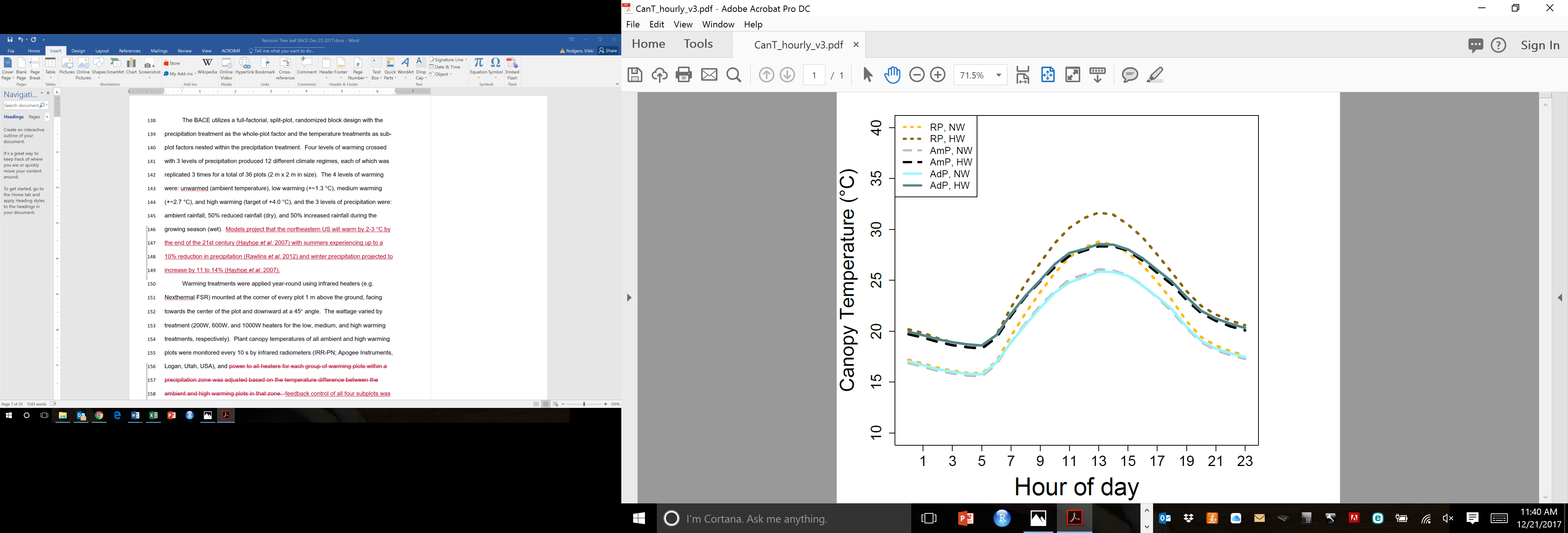


**Figure S2.** Estimated total leaf area in late July (± standard error) for all species averaged together in the wet (dark grey triangles), ambient (grey circles), and dry (light grey squares) precipitation treatments across the four warming treatments.


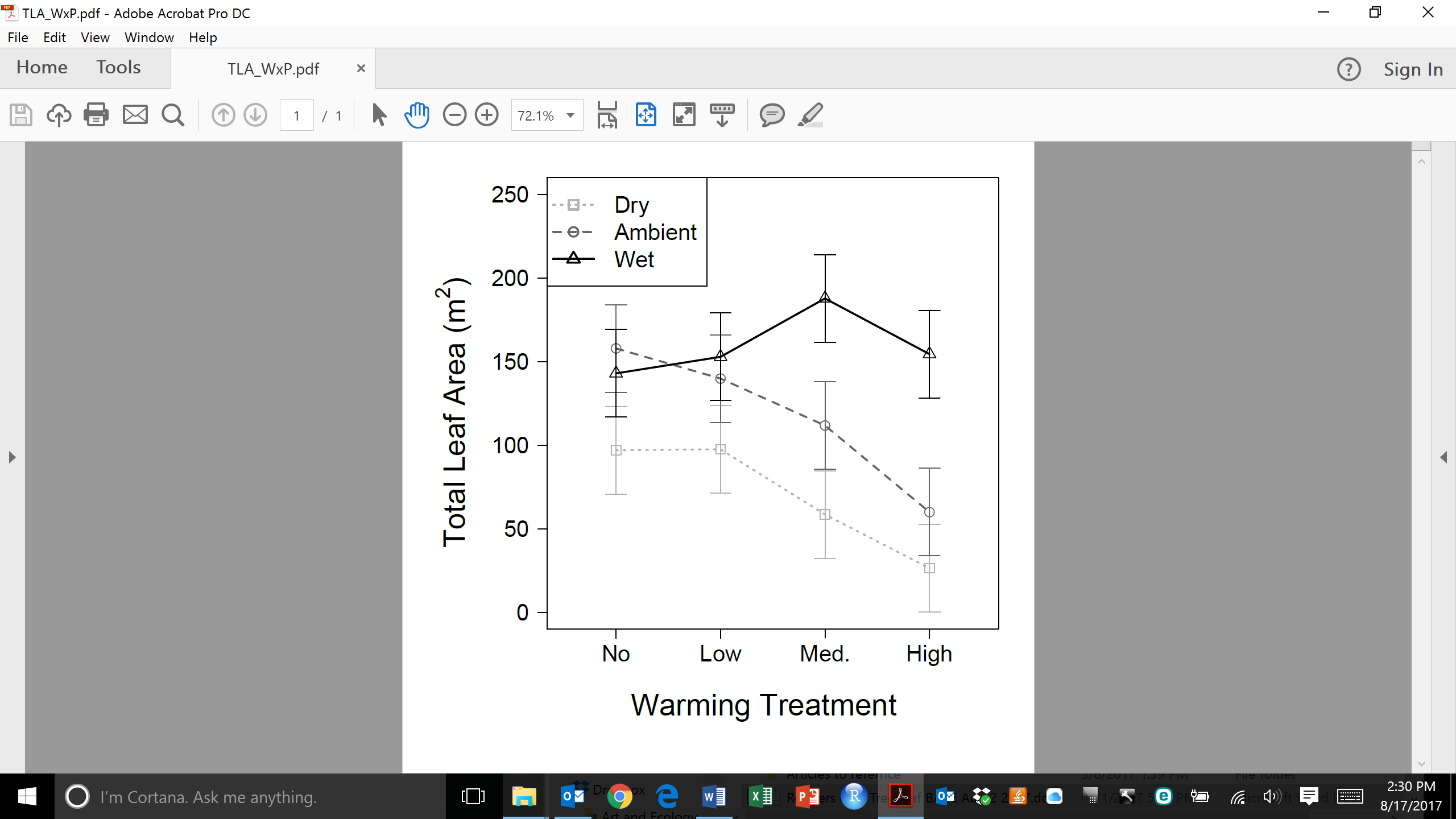


**Figure S3.** Leaf level insect herbivory (percent; mean ± standard error) for *A. rubrum*, *B. lenta*, *P. grandidentata*, *P. serotina*, *Q. rubra*, and *U. americana* in the wet (dark triangles), ambient (grey circles), and dry (light squares) precipitation treatments across the four warming treatments measured in late July.


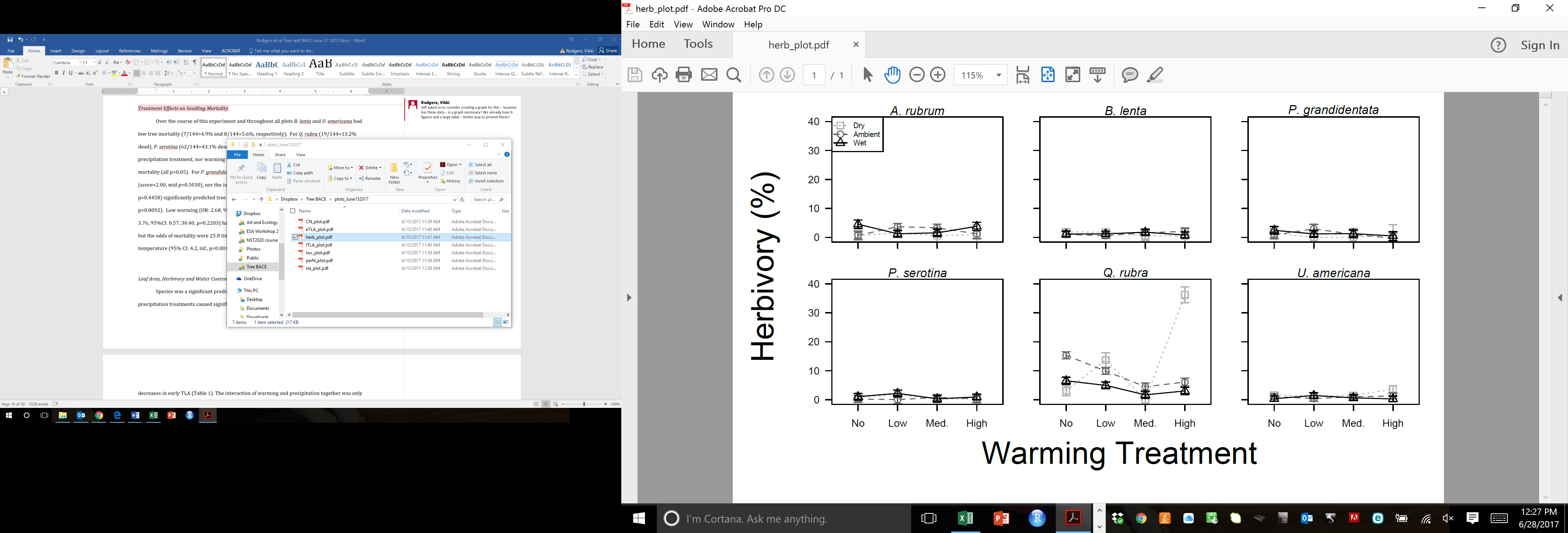


**Figure S4.** Specific leaf area (mean ± standard error) for *A. rubrum*, *B. lenta*, *P. grandidentata*, *P. serotina*, *Q. rubra*, and *U. americana* in the wet (dark triangles), ambient (grey circles), and dry (light squares) precipitation treatments across the four warming treatments measured in late July.


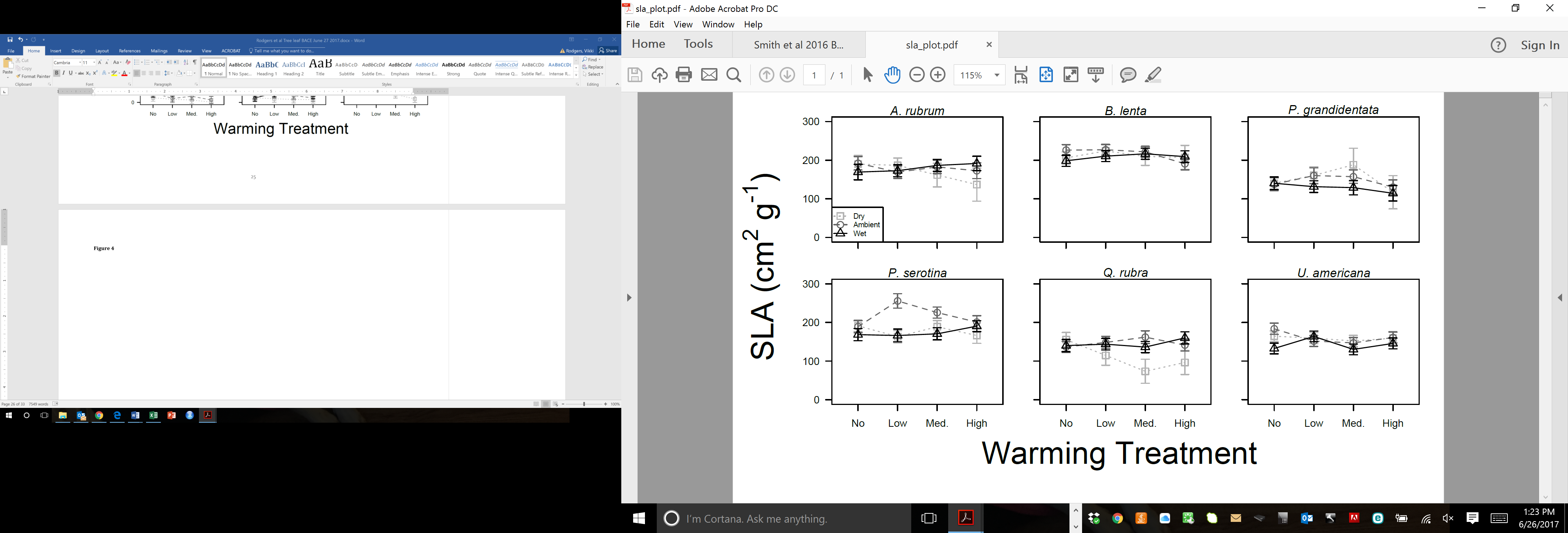


**Figure S5.** Leaf water content (percent; mean ± standard error) of *A. rubrum*, *B. lenta*, *P. grandidentata*, *P. serotina*, *Q. rubra*, and *U. americana* in the wet (dark triangles), ambient (grey circles), and dry (light squares) precipitation treatments across the four warming treatments measured in late July.


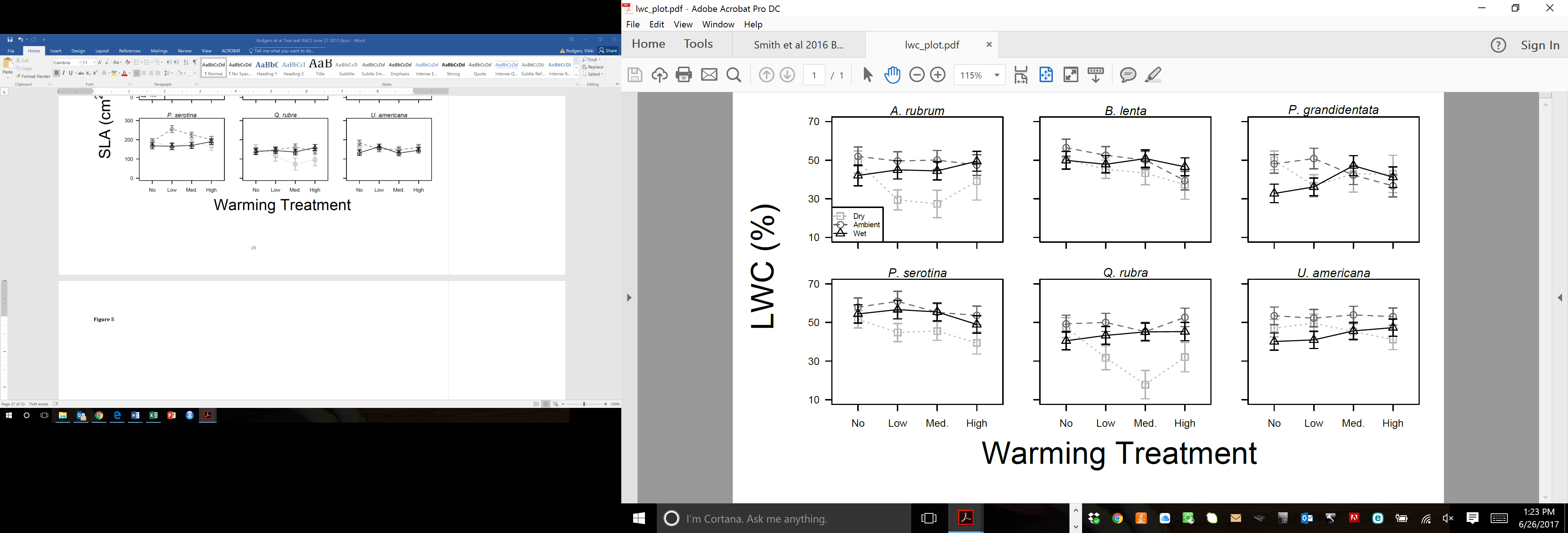


**Figure S6.** Foliar carbon by weight (percent; mean ± standard error) of *A. rubrum*, *B. lenta*, *P. grandidentata*, *P. serotina*, *Q. rubra*, and *U. americana* in the wet (dark triangles), ambient (grey circles), and dry (light squares) precipitation treatments across the four warming treatments measured in late July.


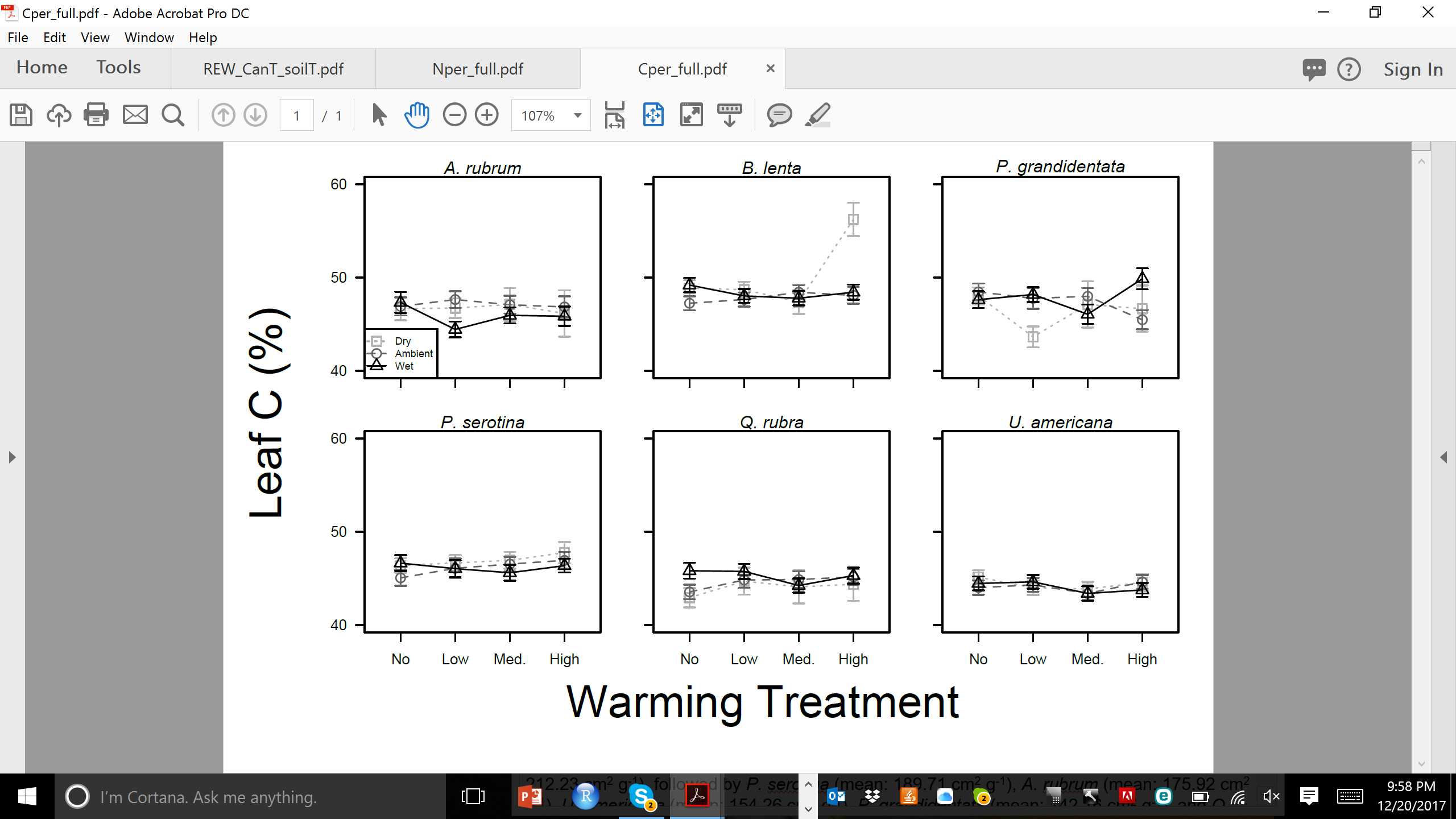

Supplement: Supporting Information [file ply003_suppl_supporting_information.docx]
